# Banana seed genetic resources for food security: Status, constraints, and future priorities

**DOI:** 10.1002/fes3.345

**Published:** 2021-11-15

**Authors:** Simon Kallow, Arne Mertens, Steven B. Janssens, Filip Vandelook, John Dickie, Rony Swennen, Bart Panis

**Affiliations:** ^1^ Royal Botanic Gardens Kew Millennium Seed Bank Ardingly UK; ^2^ Department of Biosystems Katholieke Universiteit Leuven Leuven Belgium; ^3^ Meise Botanic Garden Meise Belgium; ^4^ Biology Department Katholieke Universiteit Leuven Leuven Belgium; ^5^ International Institute of Tropical Agriculture Kampala Uganda; ^6^ Bioversity International Leuven Belgium

**Keywords:** bananas, crop wild relatives, ex situ conservation, plant genetic resources, seed banking

## Abstract

Storing seed collections of crop wild relatives, wild plant taxa genetically related to crops, is an essential component in global food security. Seed banking protects genetic resources from degradation and extinction and provides material for use by breeders. Despite being among the most important crops in the world, banana and plantain crop wild relatives are largely under‐represented in genebanks. Nevertheless, banana crop wild relative seed collections are in fact held in different countries, but these have not previously been part of reporting or analysis. To fill this gap, we firstly collated banana seed accession data from 13 institutions in 10 countries. These included 537 accessions containing an estimated 430,000 seeds of 56 species. We reviewed their taxonomic coverage and seed storage conditions including viability estimates. We found that seed accessions have low viability (25% mean) representing problems in seed storage and processing. Secondly, we surveyed 22 institutions involved in banana genetic resource conservation regarding the key constraints and knowledge gaps that institutions face related to banana seed conservation. Major constraints were identified including finding suitable material and populations to collect seeds from, lack of knowledge regarding optimal storage conditions and germination conditions. Thirdly, we carried out a conservation prioritization and gap analysis of Musaceae taxa, using established methods, to index representativeness. Overall, our conservation assessment showed that despite this extended data set banana crop wild relatives are inadequately conserved, with 51% of taxa not represented in seed collections at all; the average conservation assessment showing high priority for conservation according to the index. Finally, we provide recommendations for future collecting, research, and management, to conserve banana and plantain crop wild relatives in seed banks for future generations.

## Graphical abstract

Over 430,000 seeds of 56 banana crop wild relative taxa are stored in 13 institutions in 10 countries. Despite this, most taxa are inadequately conserved in seed banks and they remain high priority for conservation. We identify major constraints to their conservation and make recommendations for future management, research and collection.

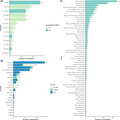

## 1 INTRODUCTION

Crop domestication is a process of genetic erosion: from wild ancestors to landraces, and to modern cultivars (Spillane & Gepts, 2000; van de Wouw et al., 2010). In the current context of anthropogenic environmental change, there is increasing need for re‐expanding crop genepools (Brozynska et al., 2016; Kersey et al., 2020; Zhang et al., 2016). At the same time, many wild ancestors, or crop wild relatives (CWRs), are extant in situ—undergoing continual evolutionary processes, such as fitness selection, environmental adaptation, and co‐evolution with pests and diseases. Genetic material in living populations therefore has adaptation potential for crops (Redden, 2013; Redden et al., 2015; Zhang et al., 2016), and its conservation is imperative for food security (Castañeda‐Álvarez et al., 2016; Dempewolf et al., 2017; Engels & Thormann, 2020).

While in situ conservation allows continued evolution and adaptation (Maxted & Kell, 2009), ex situ conservation protects genetic material from inherent in situ risks, common to wild plant species, such as genetic degradation or even extinction (Jarvis et al., 2008; Nic Lughadha et al., 2020). Seed banking is an excellent way of storing the maximum amount of genetic diversity with minimal input, for the short and potentially long term (FAO, 2014; Li & Pritchard, 2009). In addition, ex situ seed collections facilitate the relatively easy access of genetic resources for research and breeding.

The important role of CWR seed conservation is recognized in two recent major policies. The United Nations Sustainable Development Goals—Target 2, Ending Hunger, aims to, “…maintain the genetic diversity of seeds, [and] cultivated plants … and their related wild species, including through soundly managed and diversified seed and plant banks at the national, regional and international levels” (Target 2.5, UN General Assembly, 2015); and the Convention on Biological Diversity, Global Strategy for Plant Conservation, and Target 9 that aimed to conserve “…70 percent of the genetic diversity of crops including their wild relatives and other socio‐economically valuable plant species…” (CBD, 2012). It is clear, therefore, that ex situ conservation of CWRs plays an important role in addressing some of the principal challenges faced by humanity and the planet.

Bananas are one of the most important crops in the world. Approximately 114 million tons of bananas and plantains are grown and traded each year (FAO, 2019a). Alarmingly, for the many of people who rely on them, banana production is under threat by several significant diseases (Jones, 2018; Kema et al., 2021; Ploetz, 2021). On top of this, banana production is expected to be severely impacted by climate change (Brown et al., 2020; Ramirez et al., 2011; Sabiiti et al., 2018; Varma & Bebber, 2019). In this context, genetic resources contained in banana CWRs are already a promising source of disease resistance (Ahmad et al., 2020; Li et al., 2015; Rocha et al., 2021; Zuo et al., 2018) and drought tolerance for banana production (Eyland et al., 2020; Sampangi‐Ramaiah et al., 2019).

There are 108 recognized taxa in the Musaceae family (including infraspecific taxa, excluding cultivars, hereafter referred to as taxa) (Govaerts & Häkkinen, 2006; POWO, 2019). Presently, only 38 conservation assessments have been published on Musaceae taxa by the International Union for Conservation of Nature: three taxa are endangered and a further six critically so (IUCN, 2021).

The largest of the three genera in Musaceae is *Musa* L., which includes approximately 100 accepted taxa. A recent study on *Musa* found that 19% of 59 evaluated taxa were vulnerable to extinction, with an estimated 15% considered endangered (Mertens et al., 2021). *Musa* are natively distributed in tropical and subtropical Asia and the western Pacific (Govaerts & Häkkinen, 2006; Janssens et al., 2016). The second‐ largest genus in the family is *Ensete* Bruce ex Horan., containing seven taxa and distributed in tropical and southern Africa to Tropical and Subtropical Asia. Finally, the monotypic genus *Musella* is distributed in south‐central China to northern Viet Nam (Govaerts & Häkkinen, 2006).

Most of the 1000 plus, typically seedless, edible banana and plantain cultivars (hereafter referred to as “bananas”) descend from *Musa acuminata* Colla (and subspecies of) and *M*. *balbisiana* Colla (De Langhe et al., 2009; Martin et al., 2020; Perrier et al., 2011). Additionally, Fe'i bananas, eaten in Pacific regions, are likely derived from *M*. *maclayi* F. Muell. ex Mikl.‐Maclay (Ploetz et al., 2007); while *Ensete ventricosum* (Welw.) Cheesman is an important cultivated crop in Ethiopia (Borrell et al., 2019).

Most efforts in ex situ banana conservation have involved field and in vitro collections, mainly of cultivated bananas. These contain over 6600 banana accessions conserved in 31 field and in vitro collections around the world (Ruas et al., 2017). Nearly 1100 accessions are also duplicated and conserved cryogenically (van den Houwe et al., 2020), but only 163 accessions (of the 6600) are of CWRs, and when excluding *M*. *acuminata* and *M*. *balbisiana*, only 41 accessions containing 33 species remain. This means that for many taxa, there are no accessions at all, or only one single genotype is conserved (Kallow et al., 2020; Sardos, 2020; van den Houwe et al., 2020). Not only that, these conservation methods also have significant limitations: in vitro conservation is highly labor‐intensive and requires specialist laboratories and equipment, and material is also at risk of somaclonal variation and infection; field collections require considerable land, are labor‐intensive, and at risk from pests and diseases and weather events; and cryopreservation is highly labor‐intensive and requires large capital and on‐going investment (Panis et al., 2020). Seed conservation is perhaps the most efficient form of ex situ plant conservation (Liu et al., 2020).

Several recent studies have highlighted the importance of extending the diversity of banana CWRs in conservation (Castañeda‐Álvarez et al., 2016; Mertens et al., 2021; MusaNet, 2016; Sardos, 2020; van den Houwe et al., 2020). However, to date, reporting has not included extensive seed collection data. This is perhaps because many seed accessions are held in national and regional collections and not included in centralized reporting systems, or they are in storage primarily for research rather than conservation purposes. Seed conservation of banana CWRs has received less attention than living or in vitro conservation because there are presently specific constraints and limitations to it. These include: an uncertain seed storage classification (orthodox or intermediate class); loss of viability in storage; and unreliable and generally low germination rates (Kallow, Davies, et al., 2021; Kallow et al., 2020; MusaNet, 2016; Panis et al., 2020). These are exacerbated by challenges in collecting material from wild populations at full maturity. However, there has not yet been a consolidated presentation of how such constraints impact seed conservation efforts, both for institutions currently holding seed accessions, and institutions with strategic aims to seed bank bananas but who do not presently have any collections, perhaps because of the constraints mentioned.

The present study aims to (1) present the status of banana seed conservation by aggregating data from multiple institutions and countries; (2) identify the constraints to seed banking observed by a wide range of institutions across the sector; and (3) systematically assess the coverage of collections according to species distributions and provide prioritization for targeted future sampling.

## 2 METHODS

### 2.1 Accession data

The taxonomic scope of our assessment included the family Musaceae, these are CWR Taxon Groups 1–5 of bananas according to the definition of Maxted et al. (2006). To assess the status of banana seed collections, we collated accession data from multiple sources. Firstly, we gathered seed accession data from publicly available sources. We checked the US Department of Agriculture—Germplasm Resource Information Network (GRIN, https://www.ars‐grin.gov/); the Food and Agriculture Organization—World Information and Early Warning System on Plant Genetic Resources for Food and Agriculture (WIEWS, http://www.fao.org/wiews/en/); the Crop Trust—Genesys database (https://www.genesys‐pgr.org/); and the Millennium Seed Bank Partnership Data Warehouse (http://brahmsonline.kew.org/msbp/SeedData/DW). Secondly, we gathered data from a network of crop genetic resource institutions, by consulting several networks: MusaNet (https://musanet.org), the Millennium Seed Bank Partnership (MSBP, 2021), and partners involved in a running project: *BBTV mitigation*: *Community management in Nigeria*, *and screening wild banana progenitors for resistance (2015–2021)* [Bill and Melinda Gates Foundation: OPP1130226]. As a result, 22 institutions were contacted in March 2020 with a request to supply data regarding their seed accessions (institutions that supplied data and their acronyms are in Table 1). Data fields in the request were taxon name, collection source (whether seeds were collected from wild populations or cultivated plants), collection location, number of seeds, viability estimates and testing methods, number of plants sampled, storage conditions, and whether it was possible to redistribute seeds outside the institute. We did not request consent to make these data fully publicly available, so these are not presented here. If seed accessions were accessioned at the level of the hands of the infructescence (groups of fruits from the former clusters of flowers subtended by one bract, usually in two rows of fruits), we grouped them by bunch (all the seeds from the same infructescence), taking means for values. We removed duplicates and corrected synonyms using the World Checklist of the Musaceae (Govaerts & Häkkinen, 2006), with reference to other sources (The Plant List, 2013; WFO, 2021).

**TABLE 1:** List of institutions that supplied accession data

Country	Institution
Belgium (BE)	KU Leuven/Bioversity International (KUL)
Belgium (BE)	Meise Botanic Garden (MBG)
China (CN)	Germplasm Bank of Wild Species (GWS)
Great Britain (GB)	Millennium Seed Bank (MSB)
India (IN)	National Bureau of Plant Genetic Resources (NBPGR)
Indonesia (ID)	Indonesian Fruits Research Institute (ITFRI)
Indonesia (ID)	Lembaga Ilmu Pengetahuan Indonesia (LIPI)
Indonesia (ID)	Purwodadi Botanic Garden (PBG)
Malaysia (MY)	Malaysian Agricultural, Research and Development Institute (MARDI)
Nepal (NP)	National Agriculture Genetic Resource Centre (NARGC)
Philippines (PH)	National Plant Genetic Resources Laboratory (NPGRL)
Thailand (TH)	Thailand Institute of Science and Technological Research (TISTR)
Viet Nam (VN)	Plant Resources Center (PRC)

### 2.2 Constraints to banana seed banking

We conducted descriptive surveys of seed bank managers/researchers of institutions involved in ex situ banana conservation to provide a view of the constraints perceived by the sector. These included the institutions who provided accession data described above, plus 61 participants from MusaNet listed in Chase and Laliberté (2016). We highlighted three key activities of central importance to seed banking: collection, storage, and germination testing. For each of these, based on our own experience, we produced lists of potential constraints. These were then peer‐reviewed by three experts in the field, to ensure the main issues were included. Additionally, to assess the extent of experience of respondents, we included a section on “experience with seed banking activities.” Apart from this, the questions in the survey were as follows: What constraints do you have to *collecting* banana seeds? What constraints do you have to *storing* banana seeds? What constraints do you have to *germinating* banana seeds? In all cases, respondents were able to select multiple options from those provided and could also add their own additional comments. The survey was deliberately concise, with completion in 5–10 minutes, to gain maximum respondents. We produced and shared the survey using Google Forms. Respondents were given two weeks to respond to the survey in February – March 2021.

### 2.3 Gap analysis and conservation assessment

We performed a gap and conservation analysis of Musaceae taxa in ex situ seed collections by calculating four indices developed by Khoury et al. (2019). Firstly, a sampling representativeness scores for ex situ conservation (SRSex): The proportion of ex situ seed records compared with in situ occurrence records. For this index, all seed collections, including seeds from cultivated or unknown sources and those without coordinates, were used. We used the occurrence records collated and checked by Mertens et al. (2021) in the analysis. We treated *M*. *acuminata* subsp. *acuminata* and *M*. *balbisiana* var. *balbisiana* as synonyms for *M*. *acuminata* and *M*. *balbisiana* (as suggested by The Plant List, 2013). We supplemented occurrence records of these taxa and *Ensete* and *Musella* (not in the scope of Mertens et al., 2021) with more recent data (GBIF.org, 2021a, 2021b). Downloaded occurrence records were cleaned, by removing duplicated or spurious data using *CoordinateCleaner* in R (Zizka et al., 2019). Secondly, we calculated a geographic representativeness score (GRSex) and the proportion of a species distribution that seed collections are taken from. For this and the following index, we used species distribution models (SDMs) developed by Mertens et al. (2021). We only used SDMs that were significant according to Mertens et al. (2021), and only the SDM set that was not restricted according to occurrence record country or ecoregion, as some seed collections occurred beyond these areas. Models were then cropped according to species geographic distributions described by Govaerts and Häkkinen (2006). We buffered each seed collection location by a 50 km radius; and for species known to self‐pollinate indices were also calculated with a 5 km buffer, because self‐pollination restricts representativeness of *Musa* seed collections (Kallow, Panis, et al., 2021). The third index was an ecological representativeness score (ERSex). This computed the proportion of ecoregions (Olson et al., 2001) included in buffered seed collection locations to the total number of ecoregions in species SDMs. In the analysis (for GRSex and ERSex), three species SDMs were excluded as they did not overlap well with seed collection locations (*M*. *cheesmanii*, *M*.* coccinea*, and *M*. *rubra*). Several species with seed collections did not have SDMs meeting the criteria and were therefore excluded from these two index calculations but not SRSex (*M*. *halabensis*, *M*. *balbisiana* var. *andamanica*, *M*. *balbisiana* var. *liukiuensis*, *M*.* indandamanensis*, *M*.* mannii*, *M*.* muliensis*, *M*.* voonii*, all *Ensete* species, and *Musella lasiocarpa*). Collections without coordinates were geocoded using Google.com when collecting locations were described to province level or lower. Only georeferenced seed collections collected from wild populations were included in GRSex and ERSex. A final conservation score for ex situ conservation (FCSex) was calculated by the mean of the three indices (Khoury et al., 2019). Values for FCSex were then categorized as follows in the same manner as Khoury et al. (2019): <25 high priority (HP), ≥25 <50 medium priority (MP), ≥50 <75 low priority (LP), ≥75 sufficiently conserved (SC). We used all seed collections in the analysis, even those without viability estimates or those recorded as 0% viability, because only a very few viable seeds can represent the genetic diversity in *Musa* seed collections (Bawin et al., 2019; Kallow, Panis, et al., 2021), and we assumed that such low numbers may be present even in these samples. A total of 513 seed accessions of 53 taxa and 2079 occurrence records were used in the gap analysis and conservation prioritization (excluding accessions not defined to species level); of which 274 seed accessions of 26 taxa had unique coordinates and SDMs (used to calculate GRSex and ERSex). For these analyses, we used the *Gap Analysis* R package (Carver et al., 2021). To map where maximum numbers of different species could be sampled for future collection to increase GRSex and ERSex, we overlaid SDMs minus 50 km buffers of existing collecting locations and summed the number of species modelled.

## 3 RESULTS

### 3.1 Accessions

**FIGURE 1:**
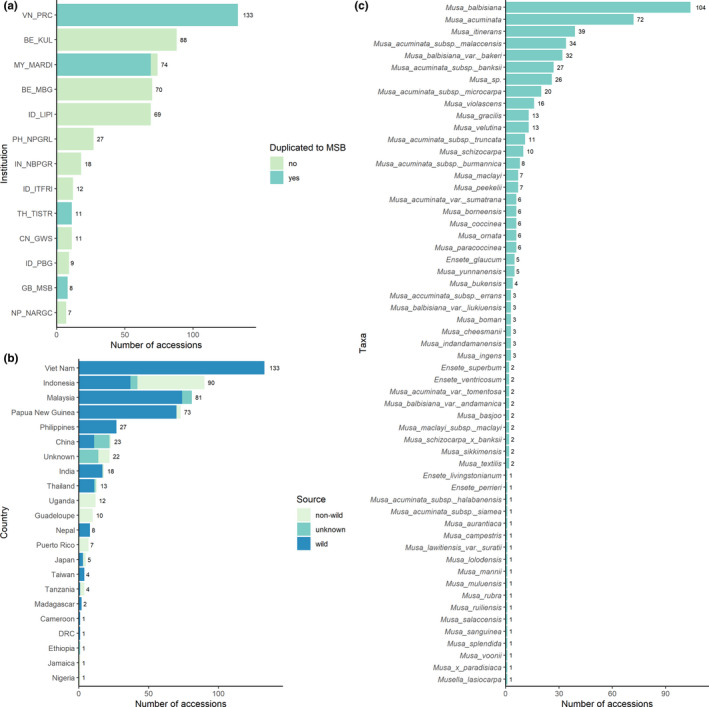
Number of seed collections and accessions according to (a) institution (see Table 1 for acronyms, MSB = Millennium Seed Bank, duplicated to MSB means some seeds from the collection are also stored at the MSB); (b) collection location; (c) taxa

From our request, we were able to collate accession data on seed collections held by 13 institutions in 10 countries (Figure 1a). In total, there were 537 accessions containing an estimated 430,000 seeds (based on a median of 800 seeds per accession). No accessions were found on GRIN or WIEWS. Accessions on Genesys and the Millennium Seed Bank Partnership Data Warehouse were duplicates of those provided by the MSB. The institution with most accessions was the PRC in Viet Nam (133 accessions), then KUL (88 accessions) and MARDI (74 accessions). Accessions at PRC, MARDI, and TISTR (plus one from GWS) were also accessioned in duplicate at the MSB, which therefore held a total of 230 accessions including these. In Indonesia, three institutions held seed accessions, and in Belgium there were two.

Seeds were collected from a total of 18 countries (Figure 1b). The country with the highest number of accessions collected was Viet Nam (133 accessions), followed by Indonesia (90 accessions), Malaysia (81 accessions), and Papua New Guinea (73 accessions); 22 accessions (4%) were of unknown collecting location. Overall, 74% of accessions were collected from wild populations, 18% from non‐wild field collections, and 7% from unknown source.

There were a total of 56 taxa included in the data (Figure 1c): 50 *Musa* taxa, five *Ensete*, and one *Musella*. Most accessions (60%) were of CWR banana Taxon Group 1b (the same species as the crop, Maxted et al., 2006). Taxon Group 1b for Fe'i bananas (*M*. *maclayi*) had a total of nine accessions; Taxon Group 1b of enset (*E*. *ventricosum*) had two accessions. Most accessions were of *M*. *balbisiana* (104 accessions) and *M*. *acuminata* (72 accessions). These included accessions not identified to subspecies level or were named *M*. *acuminata* subsp. *acuminata* or *M*. *balbisiana* var. *balbisiana* (synonyms because of unknown original publication details, The Plant List, 2013). Eighteen species (32% of species reported) were represented only by a single seed collection. There were 26 accessions (5%) identified only to the genus level (*Musa*), and some reportedly from hybrids.

Most accessions were collected between 2015 and 2018 (first quartile and third quartile respectively, median was 2016). The oldest collection was from 1967. Generally, each accession was collected from a single mother plant (median 1, first quartile 1, and third quartile 1). The number of seeds in accessions varied considerably (median 800, first quartile 200, and third quartile 2558).

Over a third of accessions (41%) were also duplicated to the MSB and were on the Genesys database (40%). In terms of availability for distribution, 55% of accessions are available, 27% are not available, and 1% are available only domestically. For 17% accessions, the distribution policy was not known.

### 3.2 Storage

**FIGURE 2:**
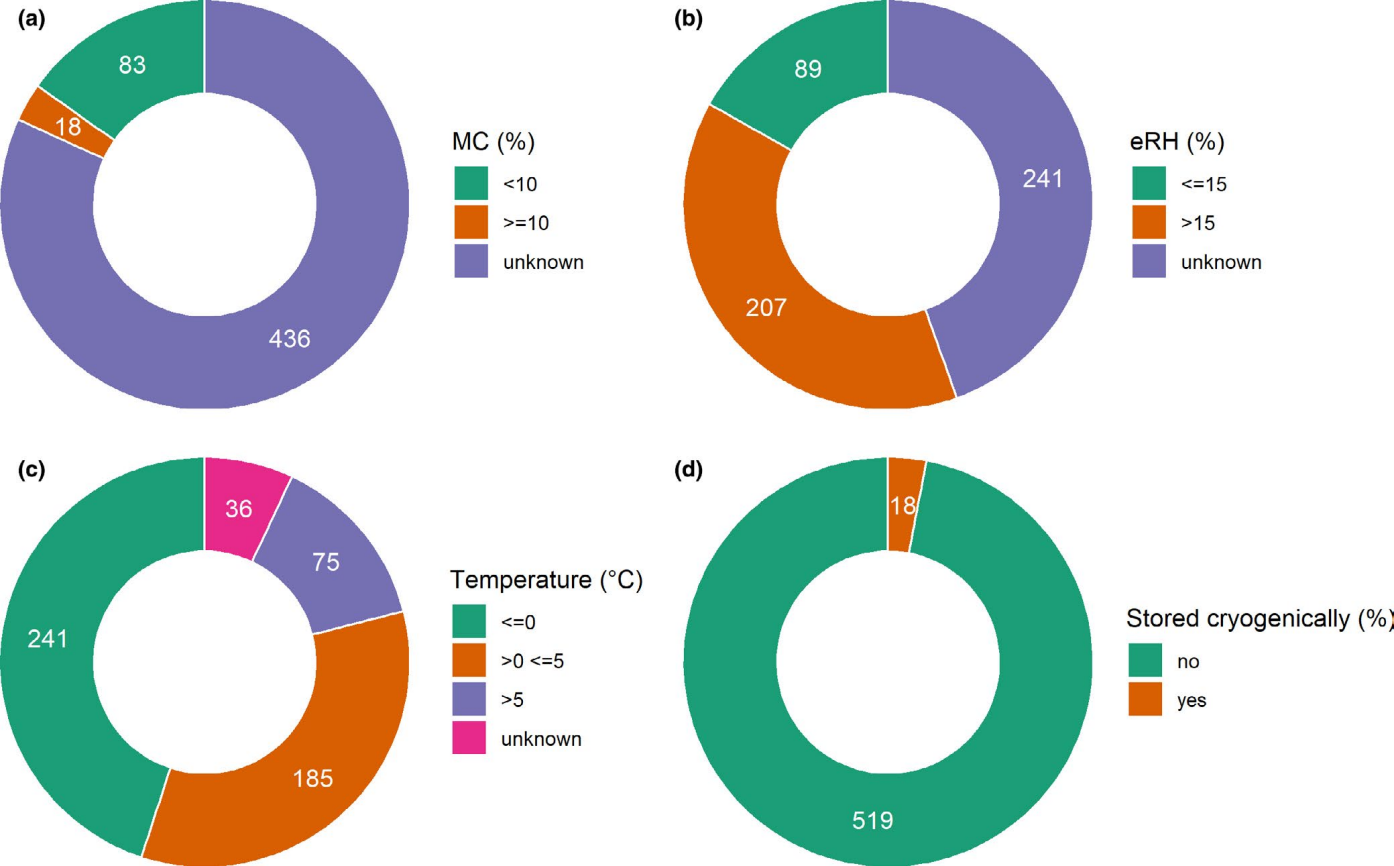
Number of accessions in seed storage conditions; (a) moisture content of seeds (MC); (b) equilibrium relative humidity (eRH); (c) storage temperature; (d) cryogenic back‐up storage; each accession counted only once (for the accessions in two conditions, long‐term storage included (−20°), medium term excluded (−5°C)

The moisture content (MC) of seeds in storage was mostly unknown (Figure 2a); the equilibrium relative humidity (eRH) of seeds was more likely to be reported than moisture content (presumably an estimation based on the relative humidity of the dry storage room). Most accessions were stored at an eRH above 15% (Figure 2b). Seeds were stored at a range of temperatures, mostly below 0°C, then in refrigerated temperatures (>0 ≤5°C), while less were stored at room temperature (>5°C, Figure 2c). Very few seeds were duplicated to cryogenic storage, in fact only accessions in India were stored in this way (Figure 2d). Apart from these, only NARGC stored seeds in more than one condition, medium term (−5°), and long term (−20°C), only two accessions were in long term, and the other five were being prepared to be placed there.

### 3.3 Viability

Median viability was 25% (first quartile 0%, third quartile 66%, excluding unknowns). More than half of accessions (52%) had not been viability assessed. Many accessions were assessed as having 0% viability (71 accessions, 13% of total). Viability may be underestimated, due to constraints in viability testing, including false negatives and unreliable methods, as discussed further below. In vitro embryo rescue (germination of the embryo extracted from the rest of the seed to remove dormancy) was the most used viability test (29% of accessions, 31 ± 31% viable), and then, it was a whole seed germination test (18% of accessions, 47 ± 37% viable). Very few were tested with the tetrazolium chloride staining test (1% of accessions, 93 ± 5% viable).

### 3.4 Perceived constraints

**FIGURE 3:**
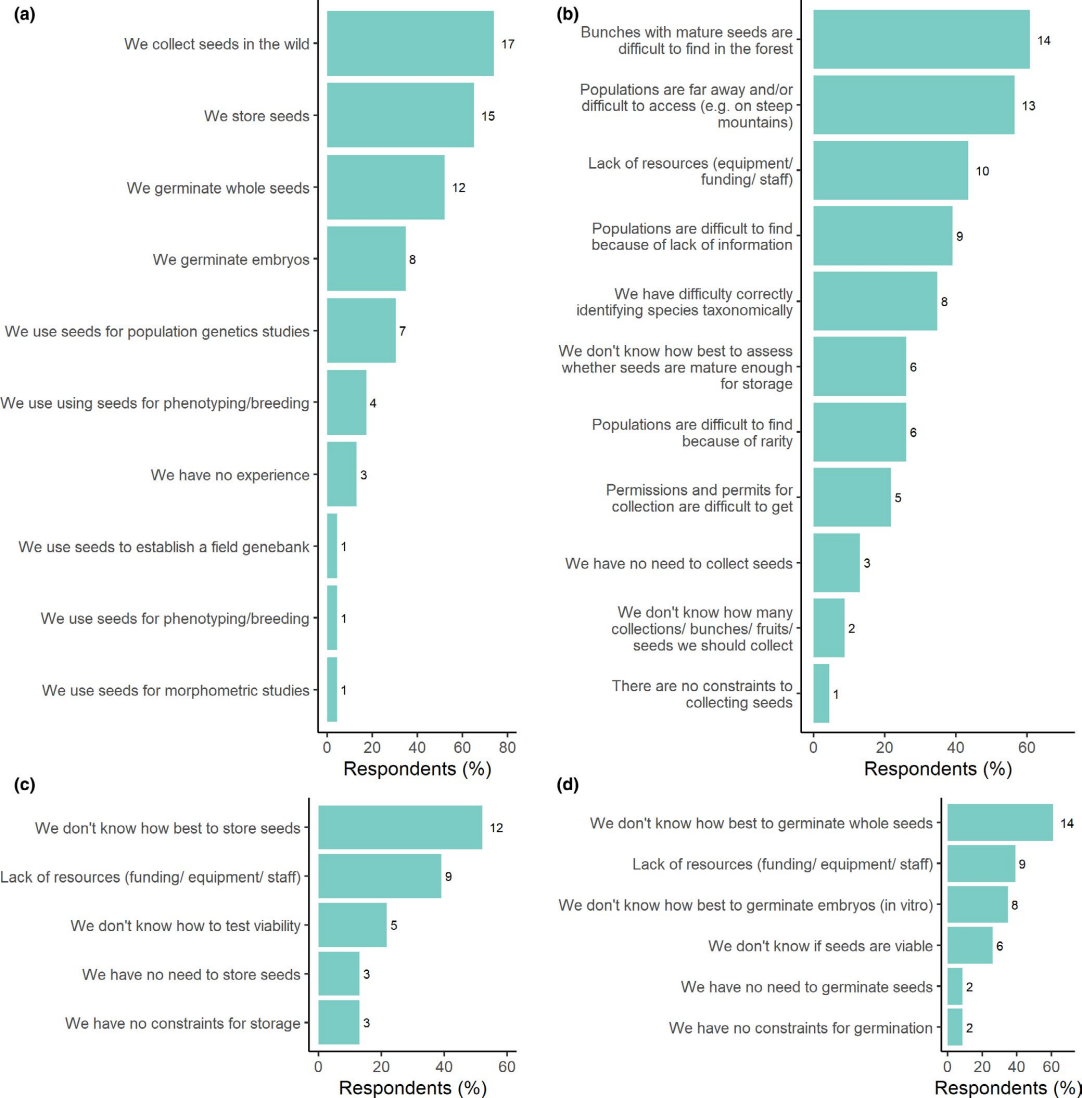
Perceived constraints to seed banking bananas, results from survey (absolute numbers shown); (a) What experience does the institution has in seed banking wild bananas? (b) What constraints are noted in *collecting* banana seeds? (c) What constraints are noted in *storing* banana seeds? (d) What constraints are noted in *germinating* banana seeds?

Our survey received 23 respondents from 22 institutions (including two from PBG). Over half of respondents (52%) also provided accession data. Respondents were involved in all areas of seed conservation (Figure 3a). Seed collecting was the primary activity of respondents (74%), followed by storage (65%) and germination (52%).

The main constraints to collecting related to challenges in accessing seeds to collect (Figure 3b). These included 61% of respondents having difficulty in finding bunches with mature seeds in the forest, and 57% of respondents highlighting the physical challenges in accessing populations due to distance or access issues. Lack of knowledge or information was a further important constraint to collectors. This included lack of distribution data for species in general (39%) and rare species in particular (26%), taxonomic difficulties (35%), and knowledge about how to assess seed maturity (26%).

**FIGURE 4:**
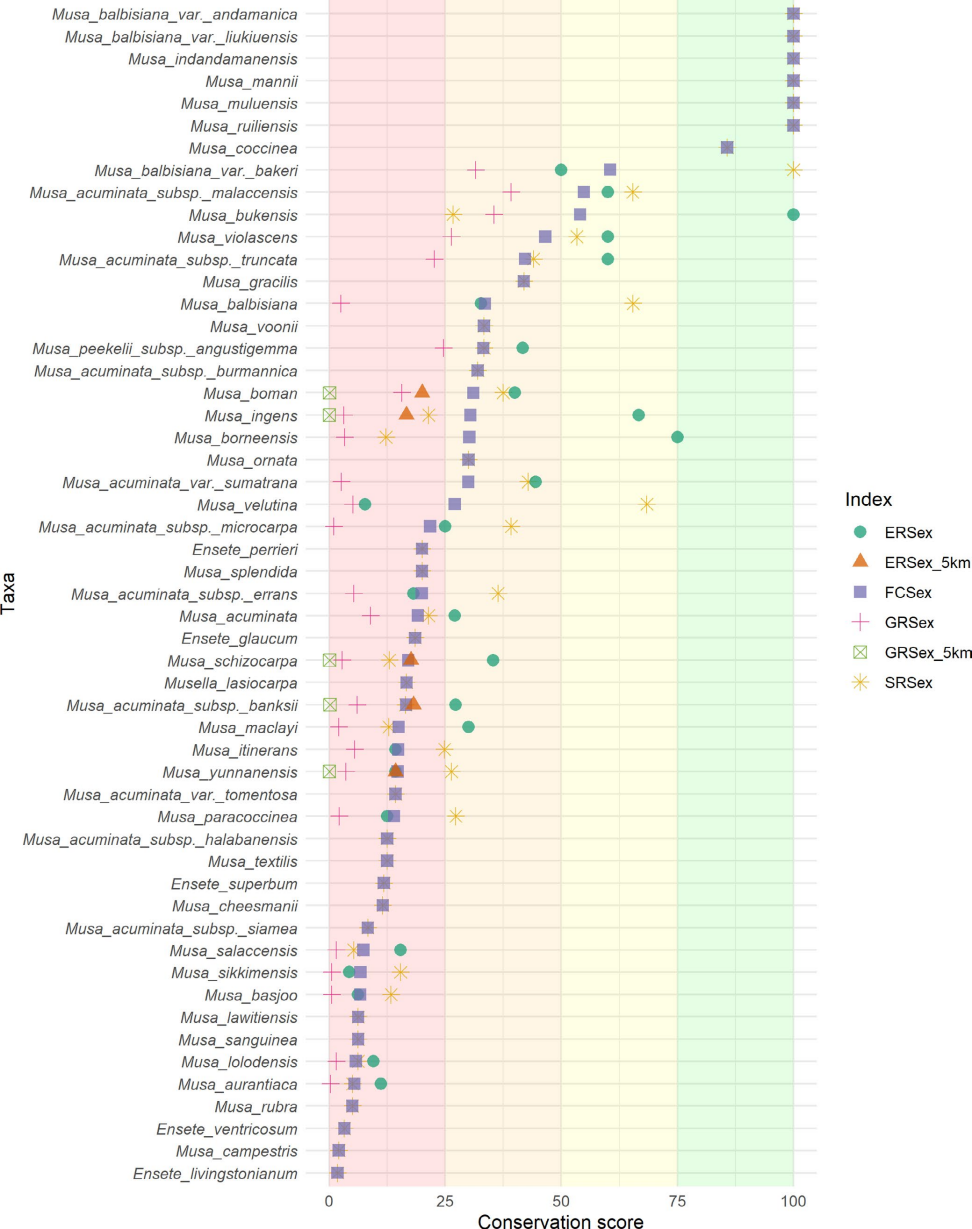
Ex situ conservation assessment of Musaceae taxa (ERSex = ecological representativeness score, FCSex = Final conservation score, GRSex = geographical representativeness score; shading represents categorization, pink = high priority, orange = medium priority, yellow = low priority, green = sufficiently conserved; values calculated with 50 km buffer of seed accessions unless stated in legend; only taxa with seed accessions shown see Figure S1 for all taxa)

Over half of respondents were constrained because they did not know how best to store banana seeds (52%) (Figure 4c). Lack of knowledge in how to assess viability of seeds in storage (22%) was also identified.

Regarding germination, the major constraint was related to lack of knowledge (Figure 3d). Firstly, this was in germinating whole seeds (61%) and then germinating embryos in vitro (35%). Again, not knowing how viable seed collections were to inform germination tests was also identified (26%).

Across all areas of seed banking, lack of resources was a key constraint (43% collecting, 39% storage, and 39% germination). Further exploration of what resources are needed is outside of the scope of the survey.

### 3.5 Conservation prioritization

The average FCSex for all taxa was 15.3, and this is categorized as HP for ex situ conservation. Overall, 85 taxa (79%) were individually categorized as HP including 55 taxa (51%) with no seed collections. Thirteen taxa (12%) were MP, three (3%) were LP, and seven (6%) were SC (Figures 4 and S1).

All SC taxa assessments relied solely on SRSex indexes, as these taxa did not have SDMs because they are recently described species with small distributions.

Additionally, *M*. *balbisiana* var. *bakeri* (LP) has a high level of sampling, but the taxonomic status of this taxon (and therefore occurrence and seed collections) is recently under question and may be more closely associated with *M*. *acuminata* (Mertens et al., 2021). *Musa bukensis* (LP), on the island of Bougainville, again has a small distribution, therefore influencing the index values.

The most well‐represented *M*. *acuminata* subspecies was *malaccensis* (LP), followed by subsp. *truncata* (MP). The least well‐represented *M*. *acuminata* taxa were subspecies *siamea* (HP), then subspecies *halabanensis* (HP). In banana CWR Taxon Group 1b, *M*. *balbisiana* was better represented than *M*. *acuminata*, with two taxa SC, one LP and one MP. *Musa balbisiana* had low GRSex because of the large distribution, but high ERSex and SRSex. *Musa maclayi* (Taxon Group 1b for F’ei banana) is HP for ex situ conservation.

No SDMs were available for *Ensete* or *Musella*, so values were based solely on SRSex. All these taxa were HP. Of these, *E*. *perrieri* received the highest FCSex, followed by *E*. *glaucum*. *Ensete ventricosum* and *E*. *livingstonianum* were among the lowest FCSex of taxa with seed collections.

When species mating systems were considered (by reducing buffer radius from 50 to 5 km), GRSex decreased and ERSex decreased by a greater extent.

### 3.6 Sampling species richness

**FIGURE 5:**
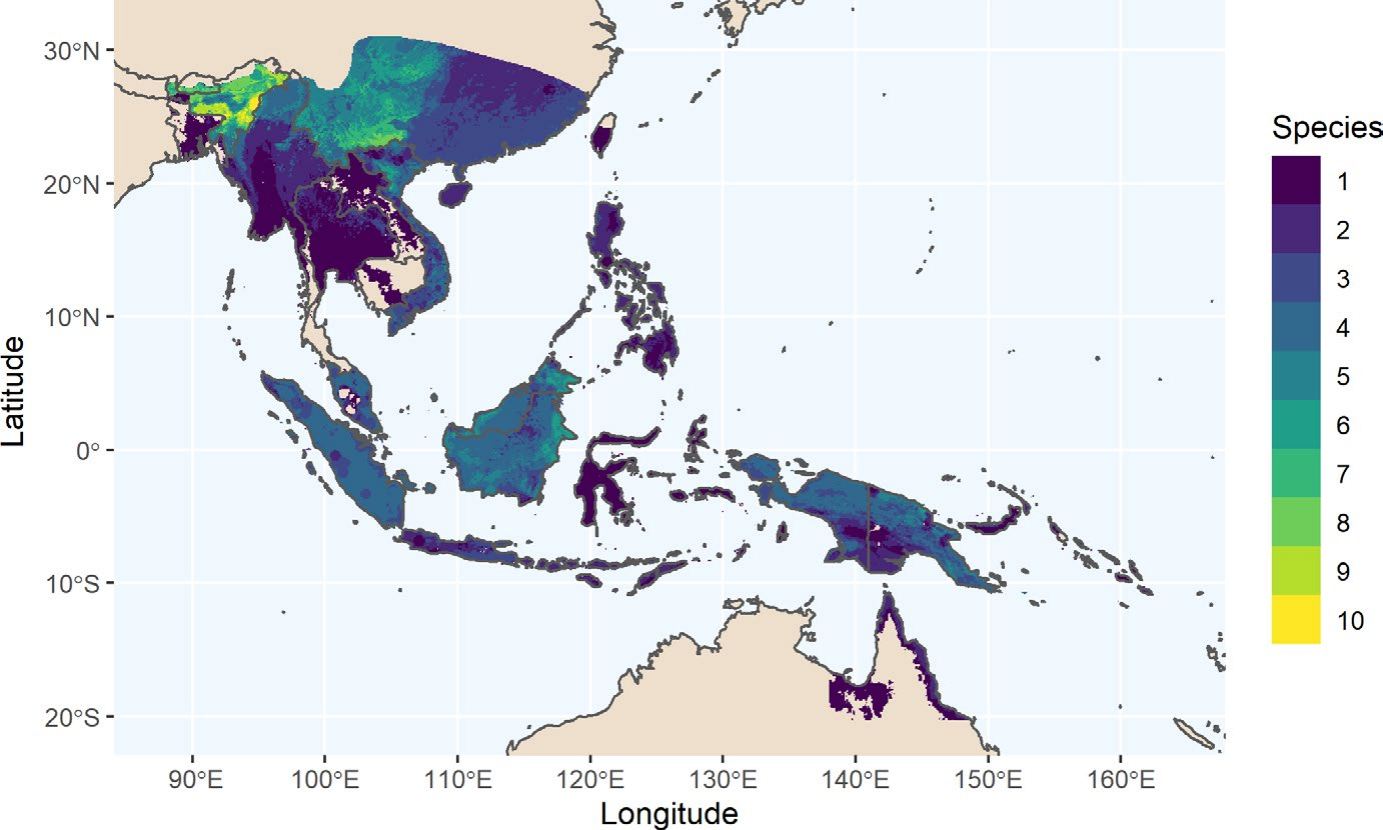
Areas with unsampled *Musa* species richness, based on overlayed SDMs (minus 50 km buffered seed collections per species, SDMs from Mertens et al. (2021)), only significant unrestricted models used, and maximum species richness per raster unit used at resolution 5′

After overlaying areas with potential banana occurrences (SDMs) where no seeds had been collected within a 50km radius, we see that the most species can be collected in the same area in NE India and Yunnan, China (Figure 5). There, up to 10 species may be collected in the same region. After that, NW Viet Nam, E and NW Borneo, and Papua New Guinea have the greatest number of unsampled taxa in the same area.

## 4 DISCUSSION

### 4.1 Status

We collated data for over 530 Musaceae seed accessions containing around 430,000 seeds, held by 13 institutions in 10 countries. We showed that many more species (56 spp.) are maintained in more seed banks (13 collections) than previously reported (Castañeda‐Álvarez et al., 2016, nine accessions of four species, and Mertens et al., 2021, 147 accessions of 13 species). In the analysis of Mertens et al. (2021), the implications of this were 46 out of 59 species did not receive an ex situ conservation assessment at all. The lack of *Ensete* accessions has previously been reported (Guzzon & Muller, 2016). Our expanded dataset increases the resolution for conservation prioritization, giving a more complete picture for targeting future seed collections. Indeed, the seed accessions presented here represent more than a threefold increase in the number of accessions of banana CWRs in storage, or several thousand‐fold increase in genotypes, as each seed is genetically unique. However, it also shows that there is much still to be done, for instance, 51% of taxa are not represented in seed collections at all, and overall, the family is HP for ex situ conservation (average FCSex = 15.3); additionally seed viability of collections is low (25% mean).

### 4.2 Status in context

Compared with other crops, banana CWRs are particularly under‐represented in genebanks. In one analysis, they were the eighth least well conserved (in situ and ex situ) among 81 crops (Castañeda‐Álvarez et al., 2016). By way of comparison, using similar methodology to the present study, wild cucurbits (*Cucurbita* L.) and sorghum are relatively better conserved both in situ and ex situ than bananas (Khoury, Carver, Kates, et al., 2020; Myrans et al., 2020), and chile peppers (*Capsicum* L.), less so, with 62% of taxa not represented in genebanks and 35 out of 37 categorized as HP (Khoury, Carver, Barchenger, et al., 2020). Additionally, in the United States, 59% of 600 CWRs recently assessed received the highest prioritization category (Khoury, Carver, Greene, et al., 2020), which is still less than Musaceae (79%).

To put this in global context, a total 5.7 million genebank accessions of plant genetic resources are presently conserved in 831 genebanks in 114 countries (FAO, 2021). Out of these, 2.8 million accessions are of CWRs, including 54,140 species of 7418 genera (i.e., 377 accessions per genus, less than the *Musa* accessions presented here). The majority of these are in long‐term (45%) or medium‐term (24%) seed storage. Many CWR seed collections were established between 2013 and 2018 as part of the Adapting Agriculture to Climate Change project, a collaboration between the Crop Trust, the Millennium Seed Bank, and 25 national partners (Global Crop Diversity Trust, 2019). During this time 4,644 seed collections were made of 371 taxa related to 28 crops, including many of those presented here. Most of these seeds are now stored at the Millennium Seed Bank.

Despite this recent upsurge in collecting, there are still considerable gaps in the genetic representativeness of many crops, with limiting factors including human capacity, facilities, funds, and management systems to target and characterize accessions (FAO, 2019b). A set of guidelines and toolkits have been developed to support the implementation of CWR conservation and sustainable use by countries and institutions (FAO, 2017; Magos Brehm et al., 2017).

### 4.3 Constraints and future priorities

#### 4.3.1 Collection

Sufficiently conserved taxa identified in the gap analysis include a few rare taxa with small distributions. This highlights the impact of targeting future collecting on the 55 *Musa* taxa for which no seed collections exist yet. Secondly, other HP taxa should be targeted to increase infraspecific diversity in collections. Thirdly, the infraspecific diversity of taxa most closely related to the edible bananas should be targeted (Taxon Group 1b—the same species as the crop). In this case, *M*. *acuminata* subspecies should be prioritized over *M*. *balbisiana*: notably, *M*. *acuminata* subsp. *siamea*, and *M*. *halabanensis*. Following this, the same section as the crop (Musa section) should be targeted using our prioritization, that is Taxon Group 2 according to Maxted at al. (2006). Furthermore, to maximize sampling efficiency (i.e. increasing GRSex in the same region), NE India and Yunnan China should be targeted for future collections.

However, as emphasized in our survey results, a lack of data on species occurrences limits collecting of target taxa. To overcome this barrier, field surveys are needed. This would improve conservation planning in general and importantly provide collectors with locations of actual populations at the fine scale from which to collect. The only way of doing this is for field missions to be carried out, in consultation with local guides, and occurrences recorded and disseminated. The sharing of occurrence records across institutions aids the accuracy and reliability of distribution information useful for all, although the wide sharing of locality information may be in conflict with in situ conservation and unregulated collection of genetic material.

While the above may improve knowledge about where to find populations, it does not meet the other key challenge: finding and collecting mature bunches in populations. Indeed, seed maturity at time of collection has a profound impact on survival and longevity of seeds in storage (Hay & Probert, 2011; Hay & Smith, 2004; Kallow et al., 2020; Singh et al., 2021). To overcome this, local people could help monitor and/or collect seeds when bunches are fully mature and protect ripening bunches from frugivores. Other options, such as creating seed orchards, may also be alternative for high‐priority species. Importantly, further research is required to evidence the most appropriate level of bunch maturity required for maximum seed survival and longevity in storage. Additionally, it may also be possible to extend the collection window by maturing bunches and their seeds after harvest (e.g., Hay & Probert, 1995).

Additionally, in our gap and conservation assessment we used GRSex and ERSex as well as taxa representativeness, importantly, these are only proxies for genetic representativeness. Assessment of genetic diversity in seed collections has been employed for only a few ex situ seed collections (Gargiulo et al., 2019; Wei & Jiang, 2020), and three *Musa* taxa (Bawin et al., 2019; Kallow, Panis, et al., 2021). These can be used as the basis of improving sampling, such as defining how many seeds or mother plants should be collected to best capture genetic diversity present in populations, and where collections should be targeted to sample uncollected alleles with optimal efficiency. Furthermore, molecular data can inform how seeds are curated and distributed, for example, how many seeds should be kept in a base collection or how many seeds should be shared with researchers to cover the diversity (Halewood et al., 2018). While this was not strongly stated in the survey, we believe that it is a priority for future seed conservation initiatives.

Furthermore, as identified in our survey, there are taxonomic needs in relation to correctly identifying samples during field missions. Training is needed in this area, but perhaps more profoundly, the genus *Musa* needs taxonomic revision (Häkkinen & Väre, 2008). Previously noted in the literature are the status of *M*. *acuminata* subspecies, notably subsp. *acuminata* and var. *tomentosa*, subsp. *errans* and subsp. *banksii* (Christelová et al., 2017); the infraspecific status of *M*. *balbisiana* taxa including subsp. *bakeri* (Mertens et al., 2021) and subsp. *andamanica* (Singh et al., 2020); the species/hybrid status of *M*. *ornata* (Christelová et al., 2017; Shepherd, 1999), and the *M*. *bukensis* and *M*. *maclayi* species complex of the Callimusa (Argent, 1976; Kallow, Panis, et al., 2021). Other areas in need of revision include the molecular delineation of newly described species (Häkkinen & Hong, 2007; Häkkinen & Teo, 2008; Häkkinen & Wang, 2008).

#### 4.3.2 Storage

Lack of knowledge about optimal seed storage conditions is a major constraint to the conservation of banana seeds. This is also evidenced by the low viability of seeds in this study (~25%). Storage conditions of accessions did not generally meet genebank standards (FAO, 2014). Only 15% of accessions were stored at <10% MC or 17% of accessions ≤15% eRH; and 45% of accessions were stored at <0°C. This means that most seeds are not stored in optimal conditions.

Recently, it was shown that when *Musa* seeds are dried to less than 10% MC, they maintain viability for at least five years without any significant loss (Panis et al., 2020), and longer term results are awaited. Panis et al. found the storage temperature was less important than seed MC for survival for this period as viability was maintained at most tested temperatures (25°C, 5°C, −20°C, and −196°C) (Panis et al., 2020). Accordingly, a storage protocol was proposed by Singh et al. (2021) advising drying *Musa* seeds to ~10% MC and storing them at −18°C to −20°C for medium term storage and −196°C for long‐term storage (Singh et al., 2021). However, data on longevity in any storage conditions beyond five years is not presently available. However, based on a review of seed longevity of 41,847 seed accessions of 276 species (Colville & Pritchard, 2019; Walters et al., 2007), one could expect Musa seeds to remain viable for several decades or longer.

Despite that, *Musa* seeds do not always maintain viability when they are dried. A recent survey found wild collected seeds were sensitive to drying (Kallow et al., 2020). Additionally, desiccation sensitivity has been shown to be related to the rate of drying and seed maturity (Singh et al., 2021). While some previous studies describe *Musa* seeds (or embryos) as having orthodox storage behavior (Chin, 1996; Panis et al., 2020; Simmonds, 1952; Singh et al., 2021; Stotzky & Cox, 1962), others describe desiccation sensitivity, being more akin to intermediate storage behavior (Abdelnouresquivel et al., 1992; Chin & Krishnapillay, 1989; Darjo & Bakry, 1990; Kallow et al., 2020; Nagano et al., 2009). Additional factors may also influence survival and longevity, such as the method of extraction from fruit pulp, post‐harvest ripening, drying rate, and intensity. Understanding and optimizing survival and longevity in storage are of upmost priority as it underpins all other efforts.

Notably, in our results, there was a lack of knowledge about the moisture content (or eRH) of the seeds in storage. Seeds were also stored in a large range of temperatures. Moreover, often the viability of seeds was not known, and sometimes, the taxonomic identity was also missing. Of course, this is because many institutions in the present study are not primarily genebank institutions. There is thus clearly scope for improving and consolidating storage conditions toward these.

Seed storage physiology is fundamental to ex situ seed conservation (Whitehouse et al., 2020), we therefore recommend a wide‐ranging assessment of Musaceae storage behavior across multiple taxa. For this, it is important that consistent methodologies are employed, for example, using the protocols as described by Hong and Ellis (1996). In addition, the same methodologies should be carried out using bunches at different maturity levels (e.g., Singh et al., 2021). This would help to identify morphological indicators of seed maturity that could be used during field missions. Key challenges for such experiments are access to suitable fresh material and consistent methodologies right through the whole process from collecting onward.

#### 4.3.3 Germination

Germination of seed collections serves two key functions: to assess the viability and longevity of collections and to access plants (for breeding, research, or regeneration of seeds) (FAO, 2014). Both functions are constrained by lack of knowledge about how to germinate seeds, as reported here. This may be overcome for *Musa* by germinating embryos extracted from seeds (i.e., embryo rescue) (Cox et al., 1960; Diro & van Staden, 2004; Pancholi et al., 1995), but this is time consuming and requires in vitro culture facilities that are not always available. Furthermore, despite being widely used by some institutions, lack of knowledge in how to germinate embryos was also emphasized as a constraint in our survey, implying that training and sharing of best practices are needed.

To optimize germination across the family, many species should be assessed with the same germination approach. To date, it appears that temperature is the primary stimulus for germination, *M*. *acuminata* and *M*. *balbisiana* seeds requiring alternating temperature regimes of around 35/20°C (Kallow, Davies, et al., 2021; Kallow, Quaghebeur, et al., 2021; Stotzky & Cox, 1962). Seeds seem to either to be non‐dormant or perhaps physiologically dormant (Chin, 1996). Dormancy is possibly removed on stratification (Kallow, Davies, et al., 2021; Kallow, Quaghebeur, et al., 2021). Further work is required in this area to develop truly reliable germination protocols. Importantly, this requires access to mature fresh material, another major constraint.

## 5 CONCLUSIONS

Conservation of banana genetic resources is globally important, and seed conservation is the most efficient way of conserving the maximum genetic diversity ex situ. We have presented a picture of the status of banana seed conservation and identified clear priorities, both for future collecting efforts and to improve the quality and management of collections. Additionally, we highlighted constraints around collecting, storing, and germinating banana seeds that must be overcome. We are convinced that coordinated efforts, systematic research, and sharing best practice are the key components to effectively conserve this valuable genetic resource for the future (Halewood et al., 2018). Coordinated joint working and sharing of experience and expertise can overcome constraints in taxonomy, for example, by effective field guides and joint field missions; in germination by embryo rescue training and research into germination eco‐physiology; viability estimates, by improving methods for the tetrazolium chloride staining test. Furthermore, it is important that reliable, consistent seed processing, drying, and storage is applied across all institutions to optimize survival and longevity.

To consolidate best practices and use of collections, we propose the development of a meta‐banana seed collection. This is similar to that for living *Musa* accessions, with the MGIS system (Ruas et al., 2017; van den Houwe et al., 2020), or the Millennium Seed Bank Partnership Data Warehouse (MSBP, 2021; Pearce et al., 2020). At present, such a system exists in the Genesys database (Data providers & the Crop Trust, 2020), however, as demonstrated in the present study, most seed collections are not part of this system. Such a meta‐collection may help to overcome some of the political and ethical barriers in relation to access and benefit sharing of plant genetic resources (Deplazes‐Zemp, 2019; Fredriksson, 2020; Neumann et al., 2018). Recent evidence suggests that such barriers are being rethought (Laird et al., 2020; Louafi & Welch, 2021; Williams et al., 2020), as the global community seeks to address shared key challenges of biodiversity loss and food security.

## AUTHOR CONTRIBUTIONS

SK involved in conceptualization, methodology, software, formal analysis, investigation, data curation, writing—original draft, writing—review and editing, and visualization. AM involved in resources, software, and data curation. SBJ involved in writing—review and editing, supervision, and funding acquisition. FV involved in writing—review and editing. JD involved in writing—review and editing, supervision, and funding acquisition. RS involved in writing—review and editing, and supervision. BP involved in conceptualization, methodology, resources, writing—review and editing, supervision, and funding acquisition.

## CONFLICT OF INTEREST

The authors declare no conflict of interest.

## Supporting information

Figure S1

## Data Availability

Research data are not shared.
