# Supplementary material for: Banana seed genetic resources for food security: Status, constraints, and future priorities
Source: Food Energy Secur. 2021 Nov 15;11(1):e345. doi: 10.1002/fes3.345 (PMC9285888; doi:10.1002/fes3.345)
Supplement: Supplementary file 1 — Figure S1 [file FES3-11-0-s001.docx]

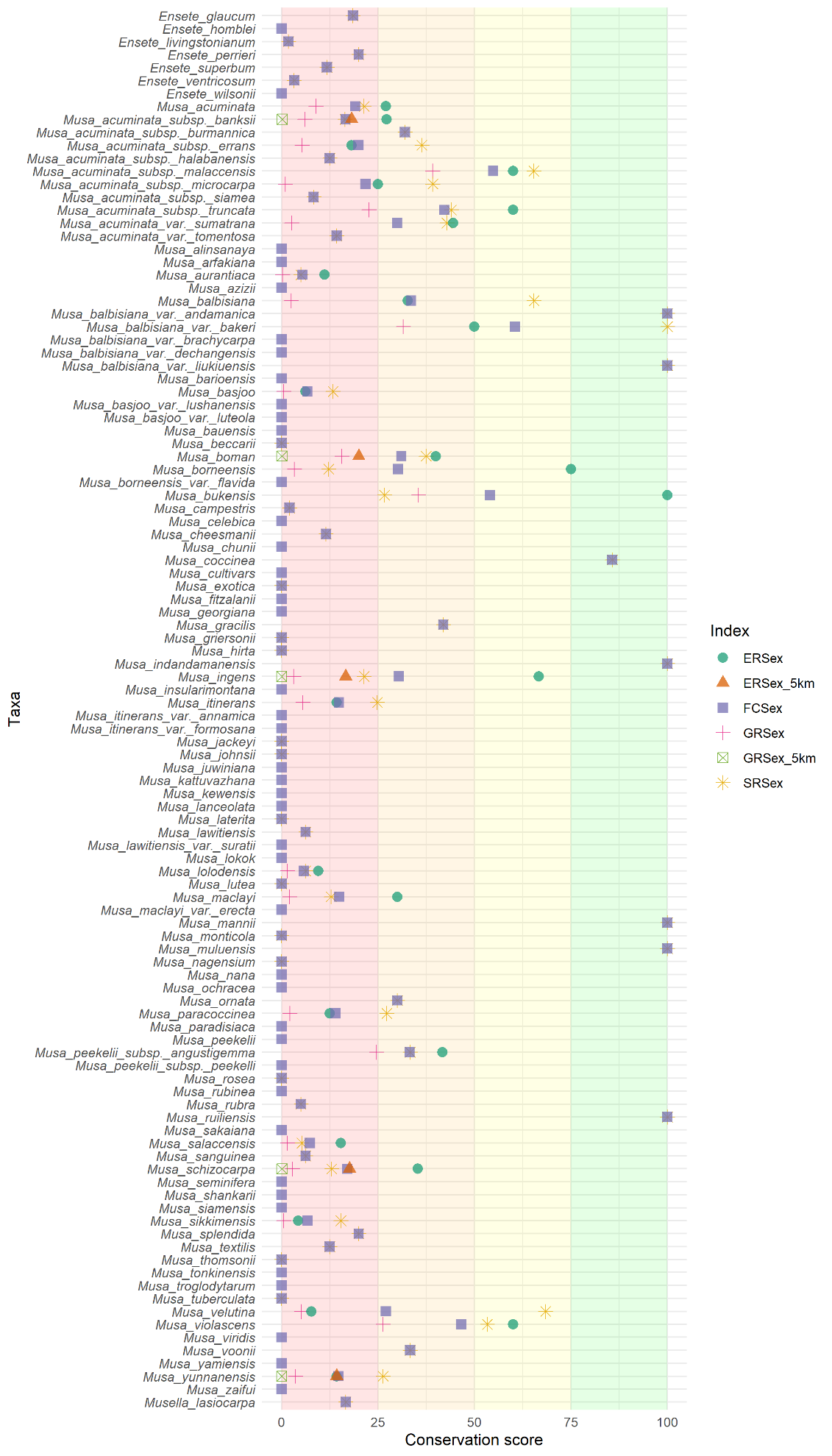


**Supplementary Figure S1.** *Ex situ* conservation assessment of Musaceae taxa, all taxa shown (ERSex=ecological representativeness score, FCSex=Final conservation score, GRSex=geographical representativeness score; shading represents categorization, pink=high priority, orange=medium priority, yellow=low priority, green=sufficiently conserved; values calculated with 50 km buffer of seed accessions unless stated in legend).
